# Reliability of Body Fat Percentage Measurements and Their Impact on Airway Resistance: Does Leptin Play a Significant Role?

**DOI:** 10.3390/medicina61081492

**Published:** 2025-08-20

**Authors:** Rodrigo Muñoz-Cofré, Edgardo Rojas-Mancilla, Pablo A. Lizana, Máximo Escobar-Cabello, Claudio García-Herrera, Daniel Conei, Fernando Valenzuela-Aedo, Francisco Javier Soto-Rodríguez, Mariano del Sol

**Affiliations:** 1 Programa de Doctorado en Ciencias Morfológicas, Universidad de La Frontera, Temuco 4811230, Chile; mariano.delsol@ufrontera.cl; 2 Unidad de Medicina Respiratoria, Departamento de Medicina Interna, Universidad de La Frontera, Temuco 4811230, Chile; 3 Unidad de Diagnóstico Laboratorio Clínico, Instituto Oncológico, Fundación Arturo López Pérez, Santiago 7500691, Chile; edgardo.rojas@falp.org; 4 Escuela de Terapia Ocupacional, Facultad de Salud y Ciencias Sociales, Universidad de Las Américas, Sede Providencia, Manuel Montt 948, Santiago 7500975, Chile; 5 Laboratory of Epidemiology and Morphological Sciences, Instituto de Biología, Pontificia Universidad Católica de Valparaíso, Valparaíso 2373223, Chile; pablo.lizana@pucv.cl; 6 Laboratorio de Función Disfunción Ventilatoria, Departamento de Kinesiología, Universidad Católica del Maule, Talca 3530000, Chile; maxfescobar@gmail.com; 7 Departamento de Ingeniería Mecánica, Universidad de Santiago de Chile, Santiago 9170022, Chile; claudio.garcia@usach.cl; 8 Departamento de Procesos Terapéuticos, Facultad de Ciencias de la Salud, Universidad Católica de Temuco, Temuco 4780000, Chile; dconei@uct.cl; 9 Escuela de Kinesiología, Facultad de Salud, Universidad Santo Tomás, Temuco 4780000, Chile; fernando.valenzuela@ufrontera.cl; 10 Departamento de Ciencias de la Rehabilitación, Facultad de Medicina, Universidad de La Frontera, Temuco 4811230, Chile; 11 Programa de Doctorado en Medicina Clínica y Salud Pública, Universidad de Granada, 18071 Granada, Spain; francisco.soto@ufrontera.cl; 12 Centro de Excelencia en Estudios Morfológicos y Quirúrgicos (CEMyQ), Facultad de Medicina, Universidad de La Frontera, Temuco 4811230, Chile

**Keywords:** validity, body fat percentage, airflow limitation, leptin

## Abstract

*Background/Objectives:* The measurement of body fat percentage (%BF) could alert us to potential respiratory problems; however, differences in %BF values have been reported depending on the method used. Therefore, the objectives of this study were to determine whether there are differences in %BF obtained through skinfold measurements (SF) and bioelectrical impedance analysis (BIA), and their correlation with airflow limitation (AFL), and secondly, to observe the relationship between leptin and AFL. *Materials and Methods:* A cross-sectional study was conducted with 80 participants (40 men and 40 women, aged 18–30 years). Assessments of %BF were made using SF and BIA. Spirometric parameters and pulmonary volumes were measured. Plasma leptin levels were determined using ELISA. Bivariate correlations and gender differences were analyzed. *Results:* When comparing %BF measured by SF and BIA, no significant differences were found between the two methods in either females or males. Furthermore, in both men and women, there was a direct and significant correlation between %BF obtained through BIA and SF (r = 0.936; r = 0.789, *p* < 0.001, respectively). Leptin showed a significant correlation with airway resistance (Raw) and specific airway resistance (sRaw) in men (r = 0.506; r = 0.553, *p* < 0.001, respectively) and women (r = 0.537, *p* = 0.001; r = 0.489, *p* = 0.003, respectively). Leptin also showed a significant correlation with %BF measured by both SF and BIA in men (r = 0.675; r = 0.687, *p* < 0.001, respectively) and women (r = 0.583; r = 0.682, *p* < 0.001, respectively). *Conclusions:* BIA and SF offer comparable results in estimating %BF. The significant correlation between leptin, %BF, and FEV1/FVC suggests a possible pathophysiological mechanism mediated by adiposity that could affect pulmonary function even in young and clinically healthy individuals.

## 1. Introduction

Currently, obesity is one of the main global health issues. In 2022, 2.5 billion adults (18 years and older) were overweight, of which 890 million were classified as obese [1]. In Chile, according to data from the Organization for Economic Co-operation and Development (OECD), 74% of the Chilean population over the age of 15 is overweight or obese. In this context, the latest National Health Survey of Chile reports that 26% of men and 32% of women are obese to varying degrees.

Obesity is defined as an “abnormal and excessive accumulation of body fat”, which has been shown to have significant effects on human health [1]. This excess body fat leads to a range of issues, from cardiovascular to motor disorders [2,3]. A less explored, yet relevant, aspect of its systemic impact is its effect on the respiratory system [3,4]. The accumulation of adipose tissue in the thoracic cavity, along with induced inflammation, may elevate leptin levels [4,5,6], which in turn could increase the activation of specific receptors in the airways, contributing to airflow limitation (AFL) [7,8].

Regarding AFL, one of the most widely studied and easily accessible variables is the forced expiratory volume in the first second (FEV_1_) [9]. However, in individuals with varying body composition and no history of respiratory disease, it would be useful to include the forced expiratory flow between 25% and 75% of the pulmonary volume (FEF_25–75_) and airway resistance (Raw) to detect respiratory problems at an early stage and prevent their progression to chronic conditions. These variables provide key information about the behavior of small airways, allowing for a more accurate assessment of pulmonary function.

In this context, measuring body fat percentage (%BF) could help alert us to potential respiratory problems [7,10]. One of the most commonly used methods is skinfold thickness (SF), due to its high clinical applicability and low cost [11]. Along the same lines, bioelectrical impedance analysis (BIA) represents a more advanced option, given the amount of information it provides and its precision, minimizing evaluator and inter-evaluator bias. Both methods are non-invasive for assessing body composition [12].

Despite its benefits, SF measurement is an indirect and user-dependent method, and therefore can be influenced by the variability of fat distribution in the human body and by the evaluator’s experience, which may lead to estimation errors [11,12]. Although BIA is more sensitive, it remains a method that requires higher-cost equipment and an appropriate setting compared to SF assessment [13]. In this context, it is reasonable to expect variations in %BF depending on the method used. The significance of this lies in the fact that variability in %BF values obtained through SF and BIA could lead to false positives in determining an individual’s body composition and, consequently, in the suspicion of potential AFL [11,12,13].

We hypothesize that a reliable measurement of %BF through skinfold assessment, when performed by a trained evaluator, allows for accurate estimation of body adiposity, showing high concordance with values obtained via BIA. Both methods are significantly correlated with Raw. Moreover, it is proposed that plasma leptin levels are directly associated with %BF and Raw, suggesting a pathophysiological link between adiposity and pulmonary function in young adults without respiratory pathology. Therefore, the objectives of this study were, first, to determine whether there are differences in %BF obtained by SF and BIA and their correlation with AFL, and second, to examine the relationship between leptin and AFL.

## 2. Materials and Methods

### 2.1. Participants

This cross-sectional study was approved by the Ethics Committee of the University of Santiago de Chile (14/2020) and conducted in accordance with the World Medical Association’s Code of Ethics (Declaration of Helsinki) for experiments involving human subjects. Upon inclusion, all participants received an oral explanation of the study objectives, and written informed consent was obtained. The sample size was calculated using the statistical software eNe 3.0. Based on the study by Rodríguez et al., which included 57 participants and reported a Raw of 3.8 ± 1.03 cmH_2_O·s [14], a statistical power of 80% and a significance level of 5% were determined. These parameters yielded a required sample size of 36 participants per sex. Accounting for a 10% dropout rate, a total of 40 participants per group were evaluated, resulting in a total sample of 80 participants. Inclusion criteria were as follows: (i) being over 18 and under 30 years of age, (ii) body composition determined by BMI as normal weight, overweight, or obese, (iii) having no signs of chronic and/or acute respiratory disease, and (iv) presenting normal spirometry values (FEV_1_ > 80% of predicted and FEV_1_/FVC > 0.7). Participants were excluded if they were smokers or had morphological alterations in the thorax or spinal column. Sampling was conducted in March 2020. All participants were assessed in a single morning session at the Pulmonary Function Laboratory of the Catholic University of Maule, Chile.

### 2.2. Measurements

All assessments were conducted in the morning (08:00 AM to 11:00 AM) and were divided into three blocks. The first block consisted of blood sample collection. The second block included the following: (i) weight and height measurements, (ii) spirometry, and (iii) lung volumes. The third block involved a cross-evaluation of the same participant using both SF and BIA.

#### 2.2.1. Anthropometry

Height was measured using a SECA^®^ stadiometer (model 220, Hamburg, Germany), recording the distance from the floor to the vertex. Participants stood upright with heels together and feet at a 45° angle. The heels, buttocks, back, and occipital region were in contact with the stadiometer. Measurement was taken during maximum inspiration while the head was aligned in the Frankfort plane. Body mass was measured using a SECA^®^ scale (model 840, Hamburg, Germany) [15]. Body mass index (BMI) was calculated by dividing weight in kilograms by height in meters squared (kg/m^2^).

#### 2.2.2. Skinfold Thickness

Adipose tissue, expressed in millimeters, was measured using a Lange Skinfold Caliper^®^ (Beta Technology, Santa Cruz, CA, USA). The skinfolds measured were as follows. Triceps: vertical fold located at the midpoint between the acromion and radial processes on the posterior aspect of the arm. Subscapular: placed 2 cm below the inferior angle of the scapula, in an oblique direction downward and laterally at a 45° angle to the horizontal. Abdominal: taken 5 cm to the right of the umbilicus, longitudinal to the midline of the body [16]. The %BF was estimated from the skinfolds using the logarithmic formula proposed by Durnin and Womersley: C − [M × log(sum of skinfolds)], where C and M are constants determined by the participant’s age and sex. This formula provides body density, which is then used in Siri’s equation: %BF = [(4.95/body density) − 4.5] × 100, to estimate total body fat percentage [10].

#### 2.2.3. Bioelectrical Impedance Analysis

To evaluate %BF using BIA, a bioelectrical impedance device was used (TANITA MC-780 MA, Tanita Corporation, Tokyo, Japan). Before assessment, participants were instructed not to wear metallic objects, avoid alcohol for 48 h, refrain from intense physical activity for 12 h, and avoid food, beverages (especially caffeine or diuretics) for 4 h. They were also instructed to urinate immediately before the evaluation [7]. The variables analyzed were weight, body fat percentage (%BF), and fat-free mass percentage (%FFM).

#### 2.2.4. Blood Plasma Sample

Blood samples were obtained in the morning by venipuncture after 8 h of fasting. The blood was collected in glass tubes containing ethylenediaminetetraacetic acid (EDTA), centrifuged at 1500× *g* for 5 min, and the plasma (supernatant) was transferred to cryotubes and stored at −80 °C until analysis [7].

#### 2.2.5. Leptin

Plasma leptin (human leptin ELISA kit, ab99978, Abcam, Cambridge, MA, USA) was measured using the ELISA technique according to the manufacturer’s instructions. Plasma samples were thawed slowly to room temperature, along with the reagents. Samples were added to wells pre-coated with anti-IL-6 and anti-leptin antibodies. After washing, a secondary antibody was added, followed by streptavidin and a second wash. The substrate was then added and incubated at room temperature to generate color. The reaction was stopped, and absorbance was measured using a spectrophotometer (Tecan, Infinite, Grödig, Austria) at 450 nm. A calibration curve was constructed, and IL-6 and leptin concentrations were calculated for each sample [7].

#### 2.2.6. Spirometry

Was performed using a body plethysmograph (MediGraphics Platinum Elite DL^®^, St. Paul, MN, USA). The highest forced vital capacity (FVC) value from three attempts that met the acceptability and reproducibility criteria established by the American Thoracic Society (ATS) was recorded. The variables used were FEV_1_ and FEF_25–75_ [17,18].

#### 2.2.7. Pulmonary Volumes

Measurements were also performed using the MediGraphics body plethysmograph (Platinum Elite DL^®^, St. Paul, MN, USA) according to ATS guidelines [19,20]. Once the cabin was sealed, participants were instructed to perform four normal tidal breaths. They were then asked to “pant gently,” aiming to move volumes between 50 and 60 mL while keeping their cheeks supported with their fingertips to minimize pressure fluctuations in the mouth. Panting was performed at a frequency of approximately 60 repetitions per minute (1 Hz). The examiner activated the shutter for 2–3 s, after which a maximal inspiration was performed, followed by expiration to residual volume (RV). The variables used were Raw and specific airway resistance (sRaw).

#### 2.2.8. Maximum Inspiratory and Expiratory Pressure (MIP–MEP)

Were assessed using the same MediGraphics body plethysmograph (Platinum Elite DL^®^, St. Paul, MN, USA). To measure MIP, participants were instructed to perform a maximal expiration, after which the pneumotachograph was occluded, and they were asked to perform a maximal inspiration against the closed valve. For MEP, participants first performed a maximal inspiration, the pneumotachograph mouthpiece was then occluded, and they were instructed to perform a maximal expiration against the closed valve. In both tests, the best result was selected from a minimum of three acceptable and reproducible attempts [20].

### 2.3. Statistical Analysis

Descriptive statistics were used to summarize the data. Statistical analysis was performed using STATA 16 (StataCorp. Stata Statistical Software, College Station, TX, USA). Data normality was assessed using the Shapiro–Wilk test. To evaluate differences in anthropometric variables, pulmonary function, and %BF between men and women, the Student’s *t*-test or the Mann–Whitney U test for independent samples was applied, depending on data distribution. To assess correlations between %BF, pulmonary function, and leptin, Pearson’s r or Spearman’s rho test was used, depending on the distribution of the variables. The intraclass correlation coefficient (ICC) was used to determine the reliability of the measurements made using the different methods used to determine %BF. Finally, a multiple linear regression was performed, considering both methods of measuring %BF separately. The dependent variable was sRAW, and the independent variables were gender. (The female gender was determined as an event of interest), age, weight, height, %BF-BIA or %BF-SF, and leptin. The stepwise method was used to determine which variables were significant in the final regression model. The level of statistical significance was set at *p* < 0.05.

## 3. Results

The total sample evaluated had an average age of 21.55 ± 2.08 years, a weight of 68.26 ± 11.76 kg, and a height of 165.0 ± 0.09 cm. The %BF-SF was 26.00 ± 8.07, and the %BF-BIA was 24.79 ± 8.66. Specifically, the results indicate that both weight and height were significantly higher in men than in women (*p* = 0.009; *p* < 0.0001, respectively). Percentage BF was significantly higher in women than in men, as measured by both SF and bioelectrical impedance BIA (*p* < 0.0001). All spirometric values and pulmonary volumes were significantly higher in men compared to women (see Table 1). Raw was significantly higher in women than in men (*p* = 0.0039). MIP and MEP were significantly higher in men compared to women (*p* = 0.0001; *p* < 0.0001, respectively). Leptin levels were significantly higher in women than in men (*p* < 0.0001) (Table 1).

**Figure 1:**
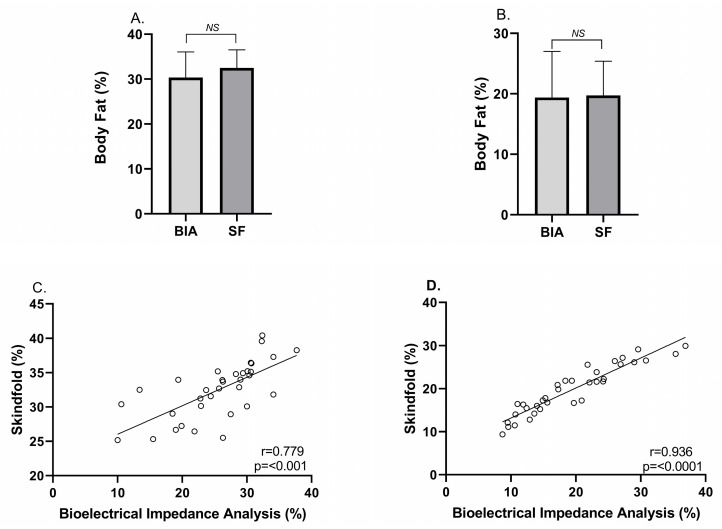
Analysis of body fat percentage by measurement method. (**A**) Comparison of body fat percentage obtained by skinfold measurement and bioelectrical impedance analysis in women. (**B**) Comparison of body fat percentage obtained by skinfold measurement and bioelectrical impedance analysis in men. (**C**) Relationship of body fat percentage between skinfold measurement and bioelectrical impedance analysis in women. (**D**) Relationship of body fat percentage between skinfold measurement and bioelectrical impedance analysis in men. BIA: bioelectrical impedance analysis; SF: skinfold.

When comparing %BF measured by SF and BIA, no significant differences were found between the two methods for either sex (see Figure 1). In addition, the intraclass correlation coefficient (ICC) was good in females (ICC = 0.854; *p* = 0.0001) and excellent in males (ICC = 0.946; *p* = 0.0001) (Table 2).

Moreover, in both men and women, a strong and significant correlation was observed between the %BF values obtained by BIA and SF (r = 0.936, *p* < 0.0001; r = 0.789, *p* < 0.0001, respectively) (Figure 1). Leptin showed a significant correlation with Raw and sRaw in women (r = 0.537, *p* = 0.001; r = 0.489, *p* = 0.003, respectively) (Table 3) and in men (r = 0.506, *p* = 0.001; r = 0.553, *p* = 0.0006, respectively) (Table 4). Leptin also showed a significant correlation with %BF measured by both SF and BIA in women (r = 0.583, *p* = 0.0004; r = 0.682, *p* = 0.0001, respectively) (Table 3) and in men (r = 0.675, *p* < 0.001; r = 0.687, *p* < 0.001, respectively) (Table 4).

Considering %BF-BIA within the model, female have 1.092 units of sRAW more than male. A %BF-BIA increase of one unit will increase sRAW by 0.090, and a %BF-BIA increase of one unit will increase sRAW by 0.001. Furthermore, by including %BF-SF instead of %BF-BIA, female have 1.001 units of sRAW more than male. Furthermore, a %BF-SF increase of one unit will increase sRAW by 0.091. Finally, a %BF-SF increase of one unit will increase sRAW by 0.001 (Table 4).

## 4. Discussion

The objectives of this study were to determine the relationship between %BF obtained via SF thickness and BIA with Raw, and to assess the relationship between leptin and Raw. In this regard, no statistically significant differences were found between the two measurement methods in addition to good to excellent reliability between both measurement methods. A correlation between SF and BIA in estimating %BF was confirmed, regardless of gender. Additionally, leptin also showed a significant relationship with %BF in both methods of assessment. Furthermore, leptin demonstrated a direct and significant association with Raw in both male and female. In addition to this, the female gender together with %BF-BIA and leptin showed a significant interaction in explaining the increase in sRAW. Therefore, one of the key findings of this study was the confirmation of the relationship between %BF and Raw, independent of the measurement method used, a relationship in which leptin appears to be involved—consistent with findings from previous studies [7,8].

Concerning the comparison between methods for measuring %BF, Gallardo-Wong et al. (2012) [21] evaluated the correlation of body composition measured by SF and BIA in first-year nutrition students. Their results indicated a direct and significant correlation between SF and BIA in the estimation of %BF. Moreover, they determined that there were no significant differences between the two methods when estimating %BF [21]. Similarly, Thakur et al. (2021) [22] aimed to compare %BF obtained using the skinfold thickness method at four sites (SFT) versus BIA in young women. Their findings showed %BF values of 32.79 ± 5.05% and 33.85 ± 5.32% for SFT and BIA, respectively. In addition to the lack of significant differences between these values, they observed a positive and significant correlation (*p* < 0.01) between the two methods [22]. Tornero-Aguilera et al. (2022) [13] compared %BF estimated using six skinfold thickness measurements and that obtained by BIA in both men and women. They found no significant differences in %BF obtained through SF and BIA [13]. These results are consistent with those of the present study, where, in addition to observing a good to excellent ICC (Table 2), no significant differences were found in %BF between SF and BIA, observing a direct and significant correlation between both instruments, regardless of gender (Figure 1). Therefore, in the absence of more technologically advanced instruments, an adequately applied and reliable skinfold assessment can provide accurate interpretation of %BF and its potential consequences—such as, in this specific case, increased Raw.

Another relevant finding of the present study was the significant relationship between leptin and %BF, regardless of the method used to assess body fat. In this regard, Muñoz et al. (2023) [7] investigated the relationship between plasma leptin levels and AFL in the small and medium airways of young adults based on their body composition. Their results showed a direct and significant correlation between leptin and %BF (r = 0.73; *p* < 0.01) in the total sample studied [7]. Similarly, Agbogulke et al. (2021) [23] determined plasma leptin concentrations in a sample of healthy African individuals, both obese and non-obese, without diabetes, and examined their relationship with anthropometric measures. Their results revealed a direct and significant correlation between leptin and %BF, both in the total sample (r = 0.57; *p* < 0.001) and when analyzed by sex (men: r = 0.72; *p* < 0.001; women: r = 0.33; *p* < 0.02) [24]. In this context, the evidence supporting a consistent relationship between leptin and %BF is reinforced, independent of age, or ethnicity. This association supports the hypothesis that a higher %BF is linked to elevated leptin levels, which could physiopathologically contribute to increased Raw.

A direct and significant relationship was observed between leptin and Raw. In this regard, Zaw et al. (2019) [24] investigated the relationship between anthropometric indices, serum leptin, and respiratory function in adult subjects. Their results showed that the median and interquartile range of serum leptin levels [5.8 (3.5–9.1) ng/mL] were significantly higher in the obese group compared to the non-obese group (*p* < 0.001) [1.9 (1.1–3.1) ng/mL]. Moreover, serum leptin levels were negatively and significantly correlated with spirometric parameters (FVC, FEV_1_/FVC, PEF, and FEF_25–75_) [24]. Parastesh et al. (2020) [25] investigated the effect of a period of aerobic exercise training on pulmonary function indices and serum leptin levels in obese men. Their baseline assessment showed that participants with normal weight had significantly lower serum leptin concentrations (*p* = 0.01) and better pulmonary function (*p* = 0.016) compared to obese participants. Additionally, they observed that leptin and obesity were inversely and significantly correlated (*p* ≤ 0.05) with FVC and FEV_1_ [25]. Although in our case no significant associations were found between leptin levels and traditional spirometric values, the observed direct and significant relationship between leptin and airway resistance indices (sRaw and Raw) suggests a potentially different mechanism of action, in which leptin may be modulating respiratory mechanics through increased airway resistance, rather than through alterations in central ventilatory capacity. These findings open new avenues for research on the role of adipose tissue–associated inflammatory mediators, such as leptin, in ventilatory function, especially in populations with excess adiposity. We believe this may be partly explained by a statistical factor: the present study opted for a sex-stratified analysis, which reduced the number of participants in each subgroup for statistical testing.

At this point, we consider it important to emphasize that statistical analyses should account for gender, given the well-documented differences in both lung function and fat distribution between male and female. Multiple logistic regression analysis showed the existence of an interaction between %BF and leptin on sRAW (Table 5), which was greater in the female gender. In this regard, leptin is known to be a peptide hormone secreted primarily by adipose tissue. In addition to modulating food intake, it also has effects on body weight and immune function. Its levels increase proportionally to fat mass. It has been shown that gender and age determine its expression level, with it being significantly higher in adult female [7,26]. In this context, leptin testing is suggested in adult female with weight disorders, considering the potential impact on the airways.

This study has several limitations that should be acknowledged: (i) the use of the gold standard method, DEXA, would have strengthened the data analysis; (ii) in clinical application, the determination of leptin would have a high economic cost; (iii) the age range of the participants was limited—expanding it to include a broader representation across the lifespan would improve generalizability; and (iv) dividing the sample according to nutritional status categories would help determine whether the observed relationships hold independently of %BF levels. Nevertheless, this study also presents several noteworthy methodological strengths. First, the assessment of %BF using two validated indirect methods under standardized conditions and in the same individuals allowed for a meaningful comparison of agreement between the procedures, providing practical evidence for clinical application. Second, the inclusion of pulmonary function measurements via spirometry and plethysmography, following international ATS/ERS guidelines, ensures high-quality data on airway resistance. Third, the analysis of the biochemical profile through plasma leptin measurement enables the exploration of pathophysiological mechanisms linking adiposity with respiratory function. Lastly, the inclusion of a sex-balanced sample strengthens comparative analyses and makes it possible to observe physiologically relevant differences between men and women.

## 5. Conclusions

No statistically significant differences were found when comparing %BF obtained through the two measurement methods. A correlation was observed between %BF and Raw in both sexes, regardless of the method used for body fat assessment. This relationship aligns with the observed correlations between leptin and %BF, as well as between leptin and Raw. Therefore, proper execution of skinfold measurement can provide a reliable estimate of %BF and serve as an early indicator of potential respiratory system complications. In this context, leptin monitoring is suggested in obese subjects under control as an indicator of possible respiratory disorders.

## Figures and Tables

**Table 1:** Baseline description of the study sample.

	Total Sample (80)	Female (40)	Male (40)	p Value
Age (years)	22 [18–30]	22 [18–30]	22 [18–25]	0.272 ^MW^
Weight (kg)	65 [52–104]	66.5 [52–104]	65 [52–92]	0.009 ^MW^
Height (m)	1.65 ± 0.09	1.62 ± 0.09	1.69 ± 0.08	<0.0001 ^t^
BMI (kg/m^2^)	24.9 [17–34]	25.30 [18–34]	24.62 [17–32]	0.790 ^MW^
%BF-SF	26.00 ± 8.07	32.47 ± 4.04	19.73 ± 5.66	<0.0001 ^t^
%BF-BIA	24.79 ± 8.66	30.34 ± 5.70	19.39 ± 7.60	<0.0001 ^t^
GT (%)	23.89 ± 7.84	25.53 ± 6.71	22.29 ± 8.60	0.086 ^t^
IGV	3.71 ± 3.30	2.82 ± 1.85	4.57 ± 3.11	0.128 ^t^
FVC (L)	4.52 ± 0.95	3.78 ± 0.54	5.25 ± 0.66	<0.0001 ^t^
FEV_1_ (L/s)	3.7 [2.02–4.74]	3.3 [2.02–4.37]	4.1 [3.3–4.7]	<0.0001 ^MW^
FEF_25–75_ (L/s)	4.13 ± 0.94	3.63 ± 0.68	4.61 ± 0.93	<0.0001 ^t^
PEF (L/s)	8.08 ± 1.72	6.75 ± 0.90	9.38 ± 1.28	<0.0001 ^t^
ERV (L)	1.35 ± 0.44	1.15 ± 0.31	1.55 ± 0.46	<0.0001 ^t^
IC (L)	2.76 ± 0.79	2.32 ± 0.50	3.19 ± 0.79	<0.0001 ^t^
RV (L)	1.91 ± 0.75	1.56 ± 0.48	2.26 ± 0.81	<0.0001 ^t^
TLC (L)	5.93 ± 1.44	5.03 ± 0.75	6.81 ± 1.41	<0.0001 ^t^
sRaw (cmH_2_O*s)	3.04 [1.18–6.83]	3.61 [1.18–6.06]	2.8 [1.27–6.83]	0.398 ^MW^
Raw (cmH_2_O/L/s)	0.9 [0.3–2.7]	1.08 [0.3–2.7]	0.61 [0.3–2.1]	0.0039 ^MW^
sGaw (1/cmH_2_O*s)	0.33 [0.15–0.85]	0.27 [0.17–0.85]	0.36 [0.15–0.79]	0.343 ^MW^
Gaw (L/s/cmH_2_O)	1.08 [0.36–3.29]	0.92 [0.36–2.68]	1.55 [0.46–3.29]	0.003 ^MW^
MIP (-cmH_2_O)	102.72 ± 33.04	87.59 ± 23.90	117.4 ± 34.32	0.0001 ^t^
MEP (cmH_2_O)	98 [36–121]	86.5 [36–121]	113 [37–179]	<0.0001 ^MW^
Leptin (ng/dL)	686 [56–2105]	1280 [136–2085]	227 [56–2105]	<0.0001 ^MW^

Data are presented as mean ± standard deviation and median [interquartile range]. BMI: body mass index; %BF-SF: body fat percentage by skinfold measurements; %BF-BIA: body fat percentage by bioelectrical impedance analysis; FVC: forced vital capacity; FEV_1_: volume that has been exhaled at the end of the first second of forced expiration; FEF_25–75_: forced expiratory flow 25–75%; PEF: peak expiratory flow; L: liters; s: seconds; ERV: expiratory reserve volume; IC: inspiratory capacity; RV: residual volume; TLC: total lung capacity; Raw: airway resistance; sRaw: specific airway resistance; cmH_2_O/L/s: centimeters of water divided by liters divided by seconds; cmH_2_O*s: centimeters of water per second; MIP: maximum inspiratory pressure; cmH_2_O: centimeters of water; MEP: maximum expiratory pressure ^MW^: Mann–Whitney; ^t^: *t*-Student.

**Table 2:** Reliability test between methods to determine body fat percentage.

Gender	%BF-SF	%BF-BIA	ICC (ICC IC_95%_)	Valor *p*
Male	19.63 ± 5.66	19.39 ± 7.60	0.946 (0.893–0.973)	0.0001
Female	32.47 ± 4.04	30.34 ± 5.70	0.854 (0.708–0.927)	0.0001
Total Sample	26.00 ± 8.07	24.78 ± 8.66	0.957 (0.931–0.974)	0.0001

Values are represented as mean ± standard deviation. ICC: intraclass correlation coefficient; 95%CI: confidence interval; %BF-SF: body fat percentage by skinfold measurements; %BF-BIA: body fat percentage by bioelectrical impedance analysis.

**Table 3:** Association between serum leptin levels and pulmonary obstruction parameters in female.

	Leptin	FEV_1_	FEF_25–75_	PEF	sRaw	Raw	%BF-SF	%BF-BIA
Leptin (ng/dL)								
FEV_1_ (L/s)	−0.077 ^p^ 0.661							
FEF_25–75_ (L/s)	0.045 ^s^ 0.798	0.779 ^s^ 0.0001						
PEF (L/s)	0.036 ^p^ 0.838	0.657 ^p^ 0.0001	0.618 ^p^ 0.0001					
sRaw (cmH_2_O*s)	0.537 ^p^ 0.001	0.121 ^p^ 0.493	−0.213 ^p^ 0.226	0.825 ^s^ 0.039				
Raw (cmH_2_O/L/s)	0.489 ^p^ 0.003	−0.072 ^p^ 0.683	−0.370 ^p^ 0.030	−0.136 ^p^ 0.441	0.900 ^p^ 0.0001			
%BF-SF	0.583 ^p^ 0.0004	−0.071 ^p^ 0.694	−0.059 ^s^ 0.743	0.159 ^p^ 0.376	0.509 ^p^ 0.002	0.484 ^p^ 0.004		
%BF-BIA	0.682 ^p^ 0.0001	0.134 ^p^ 0.455	0.047 ^p^ 0.792	0.130 ^p^ 0.468	0.591 ^p^ 0.0003	0.523 ^p^ 0.001	0.779 ^p^ <0.001	

FEV_1_: volume that has been exhaled at the end of the first second of forced expiration; FEF_25–75_: forced expiratory flow 25–75%; PEF: peak expiratory flow; L: liters; s: seconds; Raw: airway resistance; sRaw: specific airway resistance; cmH_2_O/L/s: centimeters of water divided by liters divided by seconds; cmH_2_O*s: centimeters of water per second; %BF-SF: body fat percentage by skinfold measurements; %BF-BIA: body fat percentage by bioelectrical impedance analysis; ^p^: Pearson; ^s^: Spearman.

**Table 4:** Association between serum leptin levels and pulmonary obstruction parameters in male.

	Leptin	FEV_1_	FEF_25–75_	PEF	sRaw	Raw	%BF-SF	%BF-BIA
Leptin (ng/dL)								
FEV_1_ (L/s)	−0.031 ^s^ 0.859							
FEF_25–75_ (L/s)	−0.320 ^p^ 0.060	0.134 ^p^ 0.442						
PEF (L/s)	−0.200 ^p^ 0.247	0.431 ^p^ 0.009	0.359 ^p^ 0.034					
sRaw (cmH_2_O*s)	0.506 ^s^ 0.001	0.073 ^s^ 0.673	−0.185 ^s^ 0.285	−0.121 ^s^ 0.488				
Raw (cmH_2_O/L/s)	0.553 ^s^ 0.0006	−0.027 ^s^ 0.876	−0.160 ^s^ 0.357	−0.104 ^s^ 0.548	0.903 ^s^ 0.0001			
%BF-SF	0.691 ^s^ <0.001	−0.007 ^s^ 0.965	0.019 ^s^ 0.912	0.214 ^s^ 0.216	0.548 ^s^ 0.007	0.673 ^s^ <0.001		
%BF-BIA	0.687 ^s^ <0.001	−0.022 ^s^ 0.897	0.096 ^s^ 0.581	0.101 ^s^ 0.563	0.626 ^s^ <0.0001	0.739 ^s^ <0.0001	0.936 ^s^ 0.0001	

FEV_1_: volume that has been exhaled at the end of the first second of forced expiration; FEF_25–75_: forced expiratory flow 25–75%; PEF: peak expiratory flow; L: liters; s: seconds; Raw: airway resistance; sRaw: specific airway resistance; cmH_2_O/L/s: centimeters of water divided by liters divided by seconds; cmH_2_O*s: centimeters of water per second; %BF-SF: body fat percentage by skinfold measurements; %BF-BIA: body fat percentage by bioelectrical impedance analysis; ^p^: Pearson; ^s^: Spearman.

**Table 5:** Multiple linear regression for improvement in specific airway resistance.

Model						F	*p*	R^2^	Adjusted R^2^
						9.082	**<0.001**	0.468	0.416
		95% IC							
Variable	B	LL	UL	SE	*p*				
Gender (Female)	1.092	−2.211	−0.831	0.345	**<0.001**				
%BF-BIA	0.090	0.045	0.135	0.022	**<0.001**				
Leptin	0.001	0.000	0.007	0.000	**0.008**				
Constante	−0.765	−5.833	4.304	2.535	0.764				
						F	*p*	R^2^	Adjusted R^2^
		95% IC				7.240	**<0.001**	0.412	0.355
Variable	B	LL	UL	SE	*p*				
Gender (Female)	1.001	−2.776	−1.032	0.436	<0.001				
%BF-SF	0.091	0.030	0.153	0.031	**0.004**				
Leptin	0.001	0.001	0.002	0.000	**0.001**				
Constant	−1.069	245.26	335.90	22.76	**<0.001**				

%BF-SF: body fat percentage by skinfold measurements; %BF-BIA: body fat percentage by bioelectrical impedance analysis; 95% CI: 95% confidence interval; B: unstandardized beta coefficients; LL: lower limit; UL: upper limit; SE: standard error. Bold values denote *p* < 0.05.

## Data Availability

The data used in this research is available. Please send a request to Rodrigo Muñoz-Cofré.
